# Rapid diagnostic tests duo as alternative to conventional serological assays for conclusive Chagas disease diagnosis

**DOI:** 10.1371/journal.pntd.0005501

**Published:** 2017-04-03

**Authors:** Karina E. Egüez, Julio Alonso-Padilla, Carolina Terán, Zenobia Chipana, Wilson García, Faustino Torrico, Joaquim Gascon, Daniel-Franz Lozano-Beltran, María-Jesús Pinazo

**Affiliations:** 1 Departmental Reference Laboratory, Chuquisaca Departmental Health Service (SEDES-Chuquisaca), Sucre, Bolivia; 2 Barcelona Institute for Global Health (ISGlobal), Centre for Research in International Health (CRESIB), Hospital Clinic-University of Barcelona, Barcelona, Spain; 3 School of Medicine, University of San Francisco Xavier of Chuquisaca, Sucre, Bolivia; and Health Area, Simón Bolívar Andean University, Sucre, Bolivia; 4 Fundación CEADES, Cochabamba, Bolivia; McGill University, CANADA

## Abstract

Chagas disease is caused by the parasite *Trypanosoma cruzi*. It affects several million people, mainly in Latin America, and severe cardiac and/or digestive complications occur in ~30% of the chronically infected patients. Disease acute stage is mostly asymptomatic and infection goes undiagnosed. In the chronic phase direct parasite detection is hampered due to its concealed presence and diagnosis is achieved by serological methods, like ELISA or indirect hemagglutination assays. Agreement in at least two tests must be obtained due to parasite wide antigenic variability. These techniques require equipped labs and trained personnel and are not available in distant regions. As a result, many infected people often remain undiagnosed until it is too late, as the two available chemotherapies show diminished efficacy in the advanced chronic stage. Easy-to-use rapid diagnostic tests have been developed to be implemented in remote areas as an alternative to conventional tests. They do not need electricity, nor cold chain, they can return results within an hour and some even work with whole blood as sample, like Chagas Stat-Pak (ChemBio Inc.) and Chagas Detect Plus (InBIOS Inc.). Nonetheless, in order to qualify a rapidly diagnosed positive patient for treatment, conventional serological confirmation is obligatory, which might risk its start. In this study two rapid tests based on distinct antigen sets were used in parallel as a way to obtain a fast and conclusive Chagas disease diagnosis using whole blood samples. Chagas Stat-Pak and Chagas Detect Plus were validated by comparison with three conventional tests yielding 100% sensitivity and 99.3% specificity over 342 patients seeking Chagas disease diagnosis in a reference centre in Sucre (Bolivia). Combined used of RDTs in distant regions could substitute laborious conventional serology, allowing immediate treatment and favouring better adhesion to it.

## Author summary

Chagas disease is a neglected tropical disease caused by the parasite *Trypanosoma cruzi*. It affects 6–7 million people, mainly in Latin America. An initial asymptomatic phase make the infection go undiagnosed and untreated, silently progressing into its chronic stage. About 30% of the chronically infected patients end up developing life-threatening disruption of the heart and/or gut tissues. Two drugs are available but their efficacy diminishes the longer a person has been infected. Therefore, it is of paramount importance to achieve an early diagnosis to start treatment before symptoms advance. In the chronic stage parasite presence is intermittent and diagnosis is made by serological assays that detect parasite-specific immunoglobulins. Problem is that conventional serological techniques are laborious and require equipment and trained personnel unavailable in remote regions with high disease prevalence. To circumvent those inconveniences easy-to-use rapid diagnostic tests were developed. They return results within an hour and some work with whole blood as sample allowing diagnosis out of digital puncture. We propose that conventional assays could be substituted by whole blood-friendly rapid tests in order to achieve a conclusive Chagas disease diagnosis in remote regions. This would ease access to treatment increasing adherence rates to it.

## Introduction

Chagas disease is caused by infection with the kinetoplastid parasite *Trypanosoma cruzi (T*. *cruzi)*, generally transmitted by triatomine vectors that are endemic to the Americas. In recent decades, population movements from endemic to non-endemic regions and the chance of vector-independent transmission have globalized the disease impact to non-endemic countries like Spain [1].

According to WHO reports, there are 6–7 million people infected with the parasite worldwide and 25 million live in vector transmission risk areas [2]. Despite this gruelling data there are only two chemotherapies available, benznidazole and nifurtimox, and its indication is still a matter of debate [3,4]. Although highly efficacious against the acute stage, their efficacy diminishes the longer a person has been infected. Furthermore, the treatment is useless in the later stages of chronic cardiac complications, so as it can be concluded from the outcome of the BENEFIT clinical study that investigated the therapeutic capacity of benznidazole for treating chagasic patients with advanced heart damage [5]. Despite development of more efficacious and less toxic therapies is an urgent need of Chagas disease, nowadays early accessing to presently available drugs is yet a major issue. It is estimated that current chemotherapies barely reach 1% of the infected people [2,6], which is directly related to the stigma still surrounding this infection and the fact that, very usually, goes undiagnosed until it is too late.

A timely and accurate diagnosis remains a major obstacle to start treatment. Diagnosis is hampered by the disease own characteristics, depicted by its two distinct phases. Firstly, the short acute phase is mostly asymptomatic and as a consequence it is largely undiagnosed despite parasites can be detected in blood [7]. The long indeterminate stage that follows may span decades, throughout which parasite hides away and is not easily detected despite yielding a chronic infection [7]. Then, around 30–40% of those chronically infected will develop dreadful cardiac, digestive or cardiodigestive symptoms [7]. These cause massive tissue inflammation, leading to organ dysfunction and ultimately death if untreated. Thus, the earlier the diagnosis is achieved the sooner the treatment can be administered, and the more efficacious it would be.

At the chronic indeterminate stage, with scarce or no parasites circulating, diagnosis is performed serologically [8]. The most frequently used serological tests are enzyme linked immunosorbent assays (ELISAs), either based on whole parasite lysate or recombinant proteins as antigens; indirect immunofluorescence detection (IIF); and indirect hemagglutination assay (IHA). However, resources and personnel required for the performance of those tests are limited or directly non-existent in distant regions. In order to circumvent this limitation, rapid diagnostic tests (RDTs) were developed for Chagas disease detection [9]. These are easy-to-use diagnostic tools that usually provide a result read-out within an hour. They do not require electrical equipment or trained personnel for its working and are storable and functional at room temperature [9,10]. All of them fundamental features towards their implementation in remote regions, where access to/by health providers is difficult and electrical supply can be unstable or inexistent.

Amongst the American countries where Chagas disease is endemic, Bolivia registers the highest seroprevalence rate (6.1%; [11]). In Bolivia, Chagas disease conventional diagnosis is routinely made in laboratories located in larger urban sites where required facilities are available. However, most patients live in rural or peri-urban areas where there is not access to equipped labs, nor is available the trained personnel to perform and read the tests. Currently, upon guidelines established by the Chagas National Program, serodiagnosis of the disease is made first by rapid screening with the RDT Chagas Stat-Pak (CST; Chembio Inc., Medford, USA). Positive detections are then confirmed by conventional serological tests: either by ELISA based on parasite lysate antigen or by IHA [12]. Only upon confirmation of the infection by conventional serology the treatment can be started. If results from the RDT and conventional serology disagree, a third serological assay, an ELISA based on recombinant antigen, is used to tip the scales [12]. Conventional tests provide high sensitivity and specificity, but they are time-consuming, involve multiple steps, need trained personnel to perform them, and require at least two visits of the patient to the health centre to get diagnosed. Besides, conventional Chagas diagnosis demands resources (medical capacity, laboratory equipment, blood banks, cold storage) that are scarce in rural areas where the disease has a larger prevalence. There, diagnosis by RDTs can make the difference (if a diagnostic confirmation by a conventional serology was not needed to initiate therapy).

In the present study we hypothesize that implementing the synchronous use of two RDTs based on distinct antigenic sets would suffice to deliver a definitive diagnosis in a short time, accelerating patients´ access to treatment, thus resulting in a better adherence to it. With that objective, we evaluated in parallel the performance of two RDTs: CST (Chembio Inc., Medford, USA; [13]) and Chagas Detect Plus (CDP; InBios Inc., Seattle, USA; [14]). They were compared with routinely used serological tests (IHA, lysate-antigen ELISA, and recombinant-antigen ELISA).

## Methods

### Study location, participants, sampling and design

Enrollment for this prospective study was offered to all patients ≥1 and <60 years old that attended the Chuquisaca Departmental Reference Laboratory in Sucre, either under medical prescription or by personal initiative to obtain a Chagas disease diagnosis. Patients that attended the Platform for Integral Attention to the Chagas Patient in Sucre were also offered enrollment. In any case patients came from Sucre urban area which has a population of 312,024 people (152,238 men and 159,785 women). The department of Chuquisaca, of which Sucre is the capital, is populated with 413,823 people (203,607 men and 210,216 women). None of the patients enrolled had received anti-Chagas treatment before. Upon signature of the informed consent, patients voluntarily agreed to participate in the study selflessly. In total, 342 samples were collected (223 women and 119 men). Sampling was made between March and May 2014. Whole blood for the RDTs was obtained by fingertip puncture. At the same visit a larger blood volume was also obtained by venous extraction. Extracted blood was placed in 5 ml collection tubes without anti-coagulants and let to coagulate for 10 min at room temperature. Tubes were centrifuged 10 min at 3,000 rpm to segregate the sera which was then placed into appropriately labelled tubes. Serum samples were either used immediately or stored frozen until needed.

The study was a double-blind with three different observers involved: one for the CST, other for the CDP, and a third one for the conventional serological tests (IHA, ELISA based on lysate antigen, and ELISA based on recombinant antigen). None of the observers knew the results obtained by the others at any time. All tests (two RDTs and the three conventional serological assays) were assessed over the entire population enrolled (age range ≥1 and <60 years old; n = 342). Remarkably, RDTs used whole blood as sample whereas conventional tests relied on serum samples.

### Ethics

Study was reviewed and approved by the Ethical Committees of “Fundación Ciencia y Estudios Aplicados para el Desarrollo en Salud y Medio Ambiente” (CEADES) and the Department of Health Service of Chuquisaca (SEDES Chuquisaca). Similarly, the protocol was approved by the University Andina Simon Bolivar (Sucre, Chuquisaca, Bolivia), and it was sent to both the Departmental and National Chagas Programs for its acknowledgment and approval. All patients included in the study signed an informed consent. In those cases where patients were under 18 years old (legal age), inform consent was signed by the mother, father or assigned tutor.

### Conventional serological tests

Tests used in the study are those routinely performed by the National Chagas Program and by the Platform for Integral Attention to the Chagas Patient. ELISA tests were from Wiener Lab (Rosario, Argentina) and included the parasite lysate-based ELISA (Wiener v2.0) and the recombinant antigen-based ELISA (Wiener v3.0). Tests were performed following the manufacturer´s instructions.

Regarding IHA test, Chagas Polychaco kit from Lemos Laboratorios (Buenos Aires, Argentina) was used. Briefly, assay functionality is based on the ability of anti-*T*. *cruzi* immunoglobulins to induce hemagglutination of parasite pre-sensitized sheep red blood cells. Samples were tested in a 2-fold dilution range from 1/16 up to 1/1024. Sera 1/8 dilutions were incubated with non-sensitized sheep red cells as a control for cross-reactivity with heterologous antigens. Samples that induced a hemagglutination mesh over sensitized red cells at dilutions ≥1/16 were considered positive, as far as they did not cross-react with non-sensitized red blood cells at 1/8. If cross-reactivity occurred, protocol was repeated upon treating the samples with 2-mercaptoethanol following the manufacturer´s instructions.

All individuals confirmed to be positive were offered benznidazole treatment by the medical officer in the Platform for Integral Attention to the Chagas Patient in Sucre or at the Departmental Health Service if they fulfilled treatment criteria.

### Rapid diagnostic tests

Selection of RDTs for the study was based on their ability to allow diagnosis of whole blood samples, generate results within an hour, not require instrumentation for results readout, have long shelf life at room temperature, and be based on distinct sets of parasite antigens. CST (Chembio Inc.) is in use as standard tool for Chagas disease screening by the Chagas National Program since 2005. It is based on the detection of IgG specific for the *T*. *cruzi* antigens B13, 1F8 and H49/JL7 (the latter allowing the detection of IgG antibodies characteristic of the disease chronic stage; [15]). Despite it was not commercially available at Bolivia at the study time, CDP (InBios Inc.) was chosen due to its amenability to be used with whole blood samples, the fact that barely 5 μl of it were required for a viable reading, and because it has a result turnaround of about 25 min [14]. It is based on a multi-epitope antigen (ITC8.2; [16]) which contain differs in origin from that of CST.

RDTs were made immediately upon blood collection with the second blood drop attaching to manufacturers´ instructions.

### Measurement of tests performances

The study variables considered were: (i) sensitivity, defined as the percentage of positive patients correctly diagnosed in comparison with the standard; (ii) specificity, defined as the percentage of negative patients correctly diagnosed in comparison with the standard; (iii) positive predictive value, the proportion of positive patients that were really positive; (iv) negative predictive value, the proportion of negative patients that were really so; and (v) diagnostic efficiency (i.e. accuracy), the proportion of valid results amongst the whole of them, defined therefore as the proportion of individuals correctly classified. Along the study, the standards to compare with were respectively the ELISA based on recombinant antigen (Wiener ELISA v3.0), and the coordinated results from all three conventional serological tests used.

### Data analysis and statistical methods

Data from each participant in the study were entered in Microsoft Excel and analyzed with the freely available software EPIDAT (version 3.1; Sergas, Santiago de Compostela, Spain; available at http://www.sergas.es/Saude-publica/EPIDAT). The percentage of concordance between methodologies was determined with the Kappa coefficient (κ) that compares the agreement between actual and expected results, following the criteria scale of Landis and Koch [17]. In the present study, for a RDT to be considered reliable, its κ must be >0.8. A κ value >0.8 and ≤0.9 indicated a very good concordance, whereas [0.9> κ ≤1.0] was considered excellent. In all cases, the confidence interval (CI) was set at 95% and its values are provided in parenthesis where corresponding. The Z-test was used to determine the statistical level of significance at each case (*p* <0.05). Youden´s index was calculated to determine the diagnostic efficacy (accuracy) of the RDTs combination in comparison with the conventional serological tests.

## Results

### Study demographics

The study sampling, testing and analysis were made between March and July 2014. Following the inclusion and exclusion criteria, finally 342 patients (age range ≥1 and <60 years old) participated. The majority of the samples were from patients >15 years old. Mean age of participants was 32.3 year (standard deviation ±15.2). The group composed by those >15 years of age represented 86.5% of the samples (296/342). Only 18 participants (5.3%) were under 5, and the remaining 28 (8.2%) were between 5 and 14 years of age. Women were more (65.2%) compared to men (34.8%) (male/female ratio of 0.53).

### Performance of the conventional tests

First of all, IHA and the ELISA based on parasite lysate antigen (ELISA v2.0) were assessed in a one-by-one comparison with the ELISA v3.0. IHA sensitivity was 99% (CI: 98.8–99.3%; *p* < 0.05) as it reported two false negative detections with respect to the standard ELISA v3.0 (Table 1). IHA specificity was 99.3% (CI: 98.9–99.6%; *p* < 0.05) as it also reported a false positive sample. Overall, its positive and negative predictive values were respectively 99.5% (CI: 99.3–99.8%) and 98.5% (CI: 98.1–98.9%).

**Table 1:** Categorized results of conventional serological tests in comparison to Chagas Stat-Pak (CST) and Chagas Detect Plus (CDP) RDTs.

Conventional serology					RDTs		
IHA	ELISA v2.0	ELISA v3.0	No. samples	Serological status	CST	CDP	RDTs results
N	N	N	132	Negative	131	131	Negative
P	N	N	1	Negative	1	1	Negative
N	P	N	1	Negative	1	1	Negative
N	P	P	2	Positive	2	2	Positive
P	P	P	206	Positive	206	206	Positive
		Total	342		341*	341*	

*The missing sample is a triple negative patient by conventional serology that was positive with both RDTs. N, negative; P, positive.

In contrast, screening of the 342 samples with the ELISA v2.0 yielded a sensitivity of 100% (CI: 99.8–100%; *p* < 0.05). Its specificity was 99.3% (CI: 99.8–100%; *p* < 0.05), as similarly to the IHA results, it detected a false positive serum (a different one). ELISA v2.0 positive and negative predictive values were respectively 99.5% (CI: 99.3–99.8%) and 100% (CI: 99.6–100%).

### Evaluation of CST and CDP

The performance of CST and CDP was identical (Table 1). Thus, comparison with the conventional serology results yielded exactly the same results, either evaluating them individually or as a duo. Their reported sensitivity was 100% (CI: 99.8–100%; *p* < 0.05), whereas specificity was 99.3% (CI: 98.9–99.6%; *p* < 0.05). RDTs positive predictive value was 99.5% (CI: 99.3–99.8%), and their negative predictive value was 100% (CI: 99.6–100%). In comparison with the three conventional serological tests, each RDT detected the same single false positive patient that was shown to be negative in all three conventional serology tests (Table 2).

**Table 2:** Two-by-two table of Chagas disease classification as defined by using RDTs and conventional serological tests (IHA, ELISA v2.0 and ELISA v3.0).

CST and/or CDP	Conventional serological tests		
	Positive	Negative	Total
Positive	208	1	209
Negative	0	133	133
Total	208	134	342

The level of concordance of the diagnosis efficacy achieved by a combined use of the two RDTs in comparison with the use of IHA and ELISA v2.0 measured by means of the κ coefficient was 0.97 (CI: 0.94–0.99; *p* < 0.05). On the other hand, κ coefficient obtained upon comparison of RDTs combined use with the outcome of all three conventional tests was κ = 0.99 (CI: 0.98–1.00; *p* < 0.05). Regarding the diagnostic efficiency (DE) or accuracy of the RDTs, it was 99.7% when faced with the conventional serology results (CI: 99.0–100%) and reported a Youden´s index of 0.99.Therefore, the potential of the RDTs couple to correctly detect positive and negative individuals was equivalent to the methodology conventionally used.

### Serological classification of the samples

**Fig 1:**
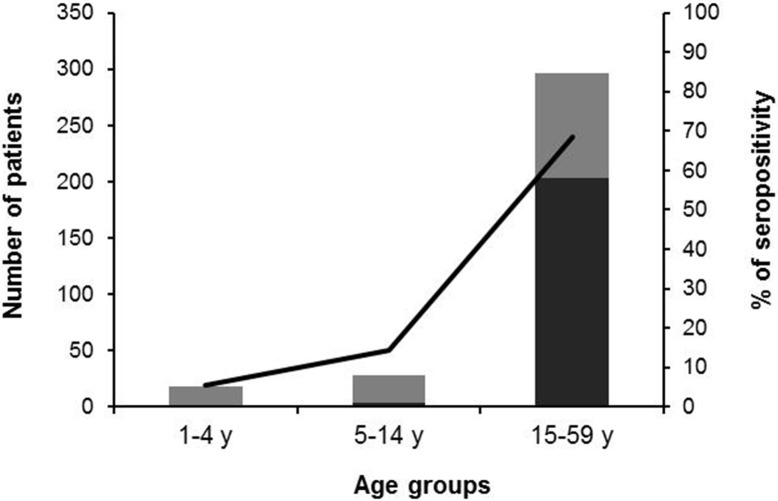
Seropositivity per age group. Serological status of patients per age group studied. Dark bars show the number of positive samples out of the total samples (whole bars) (scale at left *Y*-axis); line represents the % of seropositive patients per age group (scale at right *Y*-axis).

At the time of sampling, age and sex of the individuals participating in the study was recorded and later on used to classify the obtained results in relation to those parameters. Study included 342 individuals with ages ranging from 1 to 59 years old. If the participants´ samples are grouped in three subgroups according to their age (n_1_:1–4 years old; n_2_:5–14 years old; and n_3_:15–59 years old), the most represented is the latter (n_3_ = 296, compared to n_2_ = 28 and n_1_ = 18). It also gathers the highest percentage of seropositive individuals (68.6%, 203/296) with respect to the other two subgroups: 14.3% (4/28) and 5.6% (1/18), respectively for n_2_ and n_1_ (Fig 1). Overall, the average seroprevalence rate obtained was 60.8% (208/342).

On the other hand, regarding the sex distribution, women were more represented than men in the study (n = 342; 223 women over 119 men), and seroprevalence amongst them was higher than amongst men (women: 64.6% (144/223), versus 53.8% (64/119) of men).

## Discussion

Screening studies to validate CST and CDP have reported varied results. From the 100% agreement between ELISA and CST on almost 6,000 serum samples from Central American blood donors and Chagas patients [15], to CST 87% sensitivity and 93.2% specificity reported by Sanchez-Camargo et al. when testing sera from nine geographically apart locations [10]. In that very same study, CDP outperformed CST as it registered 93.4% sensitivity and 95.2% specificity [10]. However, CDP had averaged 84.8% sensitivity and 97.3% specificity on sera from four distinct countries (Argentina, Ecuador, Mexico and Venezuela) in another work [18]. To date, the only study that has compared CST and CDP on the same samples set, respectively determined 87.5% and 90.7% sensitivity in Bolivian sera, whilst CST and CDP sensitivity on sera from Peru was much lower (respectively, 26.6–33% and 54.3–55.2%, depending of the observer; [19]). As suggested elsewhere, it could be that in regions with lower prevalence rates, with expected lower generalized antibody titers, implementation of RDTs is not feasible at all due to very low sensitivity [20]. In both sites specificity was >98%, thus pointing at false positive detections as a cause of discordance too, which could be due to cross-reactivity with other infections, like leishmaniasis.

CST and CDP are functional as well with whole blood as sample [10,13,14], which represents a major advantage over sera as it reduces the required laboratory procedures. Besides, blood volume required for the tests is so little (10 and 5 μl respectively) that sampling can be made by digital puncture, which largely eases up the process and simplifies the screening of children. CST box includes as many Microsafe tubes (10 μL) for finger-stick whole blood acquisition as test devices, whereas CDP kit does not include them [13,14].

The first field evaluation of CST with whole blood samples (n = 1,913; all under 18 years old) from Bolivia (Sucre region) reported 93.4% sensitivity and 99% specificity (98.6% concordance between serum and whole blood results; [21]). With that sensitivity authors concluded that CST was not ideal as diagnostic tool, but claimed it was improved to 96.7% if test reading time was made at 20 min instead of the 15 min instructed by the manufacturer. We have not found such differences in our study. Considering the results of the conventional serological assays Roddy et al. found a seroprevalence rate of 6.3% (121/1913) [21]. In our case, testing a much smaller collection of patients, seroprevalence amongst those <15 years old was 11.1% (5/45). Another CST study on n = 874 blood samples from Latin American migrants in Switzerland (>16 years old; most of the infected patients were from Bolivia) determined a 95.2% sensitivity and 99.9% specificity (99.7% concordance between serum and blood samples; [22]). In agreement with that work, we have also found an excellent sensitivity (100%), a very high specificity (99.3%), and the same level of concordance between serum and blood usage with CST.

InBios CDP field evaluation with blood was also made with Bolivian samples, this time from Santa Cruz province (n = 108 adults), and from the Chaco, including pregnant women (n = 277) and children up to 17 years old (n = 200) [20]. Overall, compared to conventional serology CDP sensitivity was 96.2% and its specificity was 98.8% (95.3% serum and blood concordance; κ = 0.905). In our study, CDP also rendered excellent sensitivity and specificity (respectively, 100% and 99.3%), and the concordance between serum and blood was 99.7% (341/342; κ = 0.99). The higher seroprevalence detected by us in women (64.6%; 144/223) compared to Shah and co-workers congenital study branch (48.4%; [20]) could be due to the fact that women enrolled in their sub-study were younger in average (15–42 years old). However, within the inclusive age range between 15 and 42 years of age in our study (56.5% of women; 126/223) seroprevalence retrieved by us is still quite high (67.5%; 87/126). Thus, such difference with the previous work might be related to the locations studied and an overall higher seroprevalence in Sucre (Chuquisaca) compared to Camiri (Cordillera province, Chaco region; [20]).

Definitely, geographic variation, directly related to distinct seroprevalence rates and circulating parasite strains, is of major relevance towards RDTs implementation in the field. Therefore, in full agreement with others, RDTs must be field validated in each particular site before its use for screening is recommended [19,20]. Seroprevalence detected in this work was quite high (60.8%), but accordingly to the Bolivian government Chagas Management Departmental Program (with data from 2013), Chagas disease seroprevalence in the department of Chuquisaca was estimated to be 5.6% amongst children between 1–4 years old, 5.4% in the age range from 5 to 14 years old, and 56.6% in those ≥15 years old [23]. This is above the country´s mean (6.1%; [11]), illustrating a heterogeneous distribution of Chagas disease in the country. Although the main target population for these diagnostics are indeterminate patients, it would be interesting to address whether their performance varies in relation to the indeterminate or symptomatic stage of chronic Chagas' disease.

In the case of Bolivia, results from this study and others with Bolivian blood samples, depict CST and CDP as fast, reliable and accurate tools [19,20,22], thus bringing the chance to incorporate them in the diagnostic protocols (CST is already at the frontline for Chagas disease serological screening in Bolivia, both in blood bank screening and clinical diagnosis) [12]. However, despite RDT permeation in the health system, conventional serological diagnosis is yet necessary for infection confirmation [12,21]. This requirement for a definitive conventional diagnosis imposes a treatment start delay that can hinder patients´ adhesion to it. In the Department of Chuquisaca, most endemic municipalities present a high prevalence of *T*. *cruzi* infection and have problems to access lab services due to bad communications and lack of resources. Labs in the region are small, ill equipped, and have very limited resources. Nonetheless they are obliged by law to include Chagas disease diagnosis for the population that demands the service [24]. Taking into account that CST and CDP have shown to perform excellently in the criteria scale of Landis and Koch [17], and that they are based on distinct antigens sets, the use of the duo to substitute currently used conventional serology would be justified. Since same results were retrieved with both RDTs, it would have been very interesting to have considered other features for comparison between them (i.e. strength of signal, ease of use). However, due to the blind characteristics of the study, with a distinct observer in charge of each RDT and another one in charge of conventional serological tests, no further comparison criteria were included. Accordingly to a recent article by Angheben et al., a blind comparison of RDTs and conventional serological assays (gold-standard) should be made in a consecutive series of patients from a relevant clinical population for optimal assessment of their accuracy [25]. In view of the obtained results, the use of CST and CDP in distant regions stands as an alternative to conventional methodologies. It must be noted though that RDTs have been previously shown to lack appropriate sensitivity in regions where seroprevalence is lower than in Bolivia [19]. Since seroprevalence rates also vary between distinct regions of the country, it is inevitable to think that RDTs would perform worse than what we report here if the study had been carried out in other regions. Thus, either region-specific pilot studies or a larger study at national level including samples from geographically heterogeneous origin should be launched before fully adopting this approach.

Other issue could be RDTs´ price, which is in principle disadvantageous to that of conventional assays. In Bolivia, RDT costs range from 4$ to 7$ (US dollars) per single determination, whilst the average cost of a conventional test single readout is 1$. Nonetheless, if cost of logistics of the conventional techniques is accounted for, then their real cost is to be much higher. Despite it did not occur in this study it cannot be discarded that divergent results between both RDTs are observed. Then, a third test would still be needed for confirmation of the inconclusive outcome. Although this would increase costs, notably it should only be performed on a little percentage of expected inconclusive results and it would reasonably yet be cost-effective if conventional serology logistical costs are considered.

The retrieved results of this study validate the use of the CST and CDP diagnostic duo to reduce response time to patients and the logistic resources currently needed to elaborate a confirmed Chagas disease diagnosis. Therefore, we encourage considering the implementation of both RDTs with whole blood as sample to provide fast, reliable and definite diagnosis of Chagas disease in distant settings.

## Supporting information

S1 Checklist “STAndards for Reporting Diagnostic accuracy studies” (STARD) checklist. List of items checked towards completeness and transparency of reporting of diagnostic accuracy studies.
(DOCX)

S1 Flowchart STARD flowchart. Study flowchart depicting the final number of patients enrolled in it and their final diagnostic outcome.
(DOCX)

## References

[1] Pinazo MJ, Gascon J. Chagas disease: from Latin America to the world. Reports in Parasitology. 2015;4: 7–14.

[2] World Health Organization; Media centre; Chagas disease (American trypanosomiasis); Fact sheet updated March 2016. Available: http://www.who.int/mediacentre/factsheets/fs340/en/

[3] Sosa-Estani S, Segura EL. Etiological treatment in patients infected by *Trypanosoma cruzi*: experiences in Argentina. Curr Opin Infect Dis. 2006;19(6):583–7. doi: 10.1097/01.qco.0000247592.21295.a5 17075335 10.1097/01.qco.0000247592.21295.a5

[4] Viotti R, Alarcon de Noya B, Araujo-Jorge T, Grijalva MJ, Guhl F, Lopez MC, et al Towards a paradigm shift in the treatment of chronic Chagas disease. Antimicrob Agents Chemother. 2014;58(2):635–9. doi: 10.1128/AAC.01662-13 24247135 10.1128/AAC.01662-13 PMC3910900

[5] Morillo CA, Marin-Neto JA, Avezum A, Sosa-Estani S, Rassi A Jr., Rosas F, et al Randomized trial of benznidazole for chronic Chagas' cardiomyopathy. N Engl J Med. 2015;373(14):1295–306. doi: 10.1056/NEJMoa1507574 26323937 10.1056/NEJMoa1507574

[6] Pecoul B, Batista C, Stobbaerts E, Ribeiro I, Vilasanjuan R, Gascon J, et al The BENEFIT trial: where do we go from here? PLoS Negl Trop Dis. 2016;10(2):e0004343 doi: 10.1371/journal.pntd.0004343 26913759 10.1371/journal.pntd.0004343 PMC4767872

[7] Rassi A Jr, Rassi A, Marin-Neto JA. Chagas disease. Lancet. 2010; 375(9723):1388–402. doi: 10.1016/S0140-6736(10)60061-X 20399979 10.1016/S0140-6736(10)60061-X

[8] Otani MM, Vinelli E, Kirchhoff LV, del Pozo A, Sands A, Vercauteren G, et al WHO comparative evaluation of serologic assays for Chagas disease. Transfusion. 2009;49(6):1076–82. doi: 10.1111/j.1537-2995.2009.02107.x 19290995 10.1111/j.1537-2995.2009.02107.x

[9] Medecins Sans Frontieres. Campaign for access to essential medicines. International meeting: new diagnostic tests are urgently needed to treat patients with Chagas disease. Rev Soc Bras Med Trop. 2008;41(3):315–9. 18719818 10.1590/s0037-86822008000300020

[10] Sanchez-Camargo C, Albajar-Viñas P, Wilkins PP, Nieto J, Leiby DA, Paris L, et al Comparative evaluation of 11 commercialized rapid diagnostic tests for detecting *Trypanosoma cruzi* antibodies in serum banks in areas of endemicity and nonendemicity. J Clin Microb. 2014;52(7):2506–12. 10.1128/JCM.00144-14 PMC4097696 24808239

[11] Chagas disease in Latin America: an epidemiological update based on 2010 estimates. WHO Weekly Epidemiological Record (WER). 2015; vol.90, 6 (pp. 33–44). 25671846

[12] Manual de normas técnicas y operativas para el tamizaje, diagnóstico y tratamiento de la enfermedad de Chagas crónica reciente infantil. Serie de documentos técnicos normativos. 2ª Edición. Publicación 30. Ministerio de Salud del Estado Plurinacional de Bolivia, La Paz, Bolivia; 2007. [Cite in Spanish].

[13] http://chembio.com/products/human-diagnostics/chagas-stat-pak-rapid-assay/.

[14] http://www.inbios.com/chagas-detecttm-plus-rapid-test-intl/.

[15] Ponce C, Ponce E, Vinelli E, Montoya A, de Aguilar V, Gonzalez A, et al Validation of a rapid and reliable test for diagnosis of Chagas’ disease by detection of *Trypanosoma cruzi*-specific antibodies in blood of donors and patients in Central America. J Clin Microb. 2005;43(10):5065–8. 10.1128/JCM.43.10.5065-5068.2005 PMC1248447 16207963

[16] Houghton RL, Stevens YY, Hjerrild K, Guderian J, Okamoto M, Kabir M, et al Lateral flow immunoassay for diagnosis of *Trypanosoma cruzi* infection with high correlation to the radioimmunoprecipitation assay. Clin Vaccine Immunol. 2009;16(4):515–20. doi: 10.1128/CVI.00383-08 19211772 10.1128/CVI.00383-08 PMC2668284

[17] Landis JR, Koch GG. The measurement of observer agreement for categorical data. Biometrics. 1977; 33(1):159–74. 843571

[18] Reithinger R, Grijalva MJ, Chiriboga RF, de Noya BA, Torres JR, Pavia-Ruz N, et al Rapid detection of *Trypanosoma cruzi* in human serum by use of an immunochromatographic dipstick test. J Clin Microbiol. 2010;48(8):3003–7. doi: 10.1128/JCM.02474-09 20534801 10.1128/JCM.02474-09 PMC2916568

[19] Verani JR, Seitz A, Gilman RH, LaFuente C, Galdos-Cardenas G, Kawai V, et al Geographic variation in the sensitivity of recombinant antigen-based rapid tests for chronic *Trypanosoma cruzi* infection. Am J Trop Med Hyg. 2009;80(3):410–5. 19270291

[20] Shah V, Ferrufino L, Gilman RH, Ramirez M, Saenza E, Malaga E, et al Field evaluation of the InBios CDP rapid test in serum and whole-blood specimens in Bolivia. Clin Vaccine Immunol. 2014;21(12):1645–9. doi: 10.1128/CVI.00609-14 25274804 10.1128/CVI.00609-14 PMC4248774

[21] Roddy P, Goiri J, Flevaud L, Palma PP, Morote S, Lima N, et al Field evaluation of a rapid immunochromatographic assay for detection of *Trypanosoma cruzi* infection by use of whole blood. J Clin Microb. 2008;46(6):2022–7. 10.1128/JCM.02303-07 PMC2446863 18400910

[22] Chappuis F, Mauris A, Holst M, Albajar-Viñas P, Jannin J, Luquetti AO, et al Validation of a rapid inmunochromatographic assay for diagnosis of *Trypanosoma cruzi* infection among Latin-American migrants in Geneva, Switzerland. J Clin Microbiol. 2010;48(8):2948–52. doi: 10.1128/JCM.00774-10 20554821 10.1128/JCM.00774-10 PMC2916554

[23] Programa Nacional de Chagas. Ministerio de Salud del Estado Plurinacional de Bolivia. Informe Anual Epidemiológico. La Paz, Bolivia, 2013. [Cite in Spanish].

[24] Bolivia: Ley N° 3374, 23 de marzo de 2006. Layout (in Spanish) available at: http://www.lexivox.org/norms/BO-L-3374.xhtml. [Cite in Spanish].

[25] Angheben A, Gobbi F, Buonfrate D, Tais S, Degani M, Anselmi M, et al Notes on rapid diagnostic tests for chronic Chagas disease. Bull Soc Pathol Exot. 2017 1 23. [Epub ahead of print]. 10.1007/s13149-017-0546-6 28116568

