# Supplementary material for: Rapid diagnostic tests duo as alternative to conventional serological assays for conclusive Chagas disease diagnosis
Source: PLoS Negl Trop Dis. 2017 Apr 3;11(4):e0005501. doi: 10.1371/journal.pntd.0005501 (PMC5391121; doi:10.1371/journal.pntd.0005501)
Supplement: S1 Flowchart — Study flowchart depicting the final number of patients enrolled in it and their final diagnostic outcome. (DOCX) [file pntd.0005501.s002.docx]

**Potentially eligible participants**

**n = 377**

**Excluded**

Reason 1: already treated (n = 10)

**Eligible participants**

**n = 367**

**Excluded**

n = 25

- Reason 2: pregant (n = 3)
- Reason 3: refused to participate (n = 22)

**Participants enrolled and tested by RDTs duo (Stat-Pak/InBios)**

**n = 342**

Positive to both tests

n = 209

Indeterminate

n = 0

Negative to both tests

n = 133

Reference standard test (recombinant ELISA) was not used

n = 0

Reference standard test (recombinant ELISA) was not used

n = 0

Reference standard test (recombinant ELISA) was not used

n = 0

**Indeterminate** to the standard reference test

n = 0

**Positive** to the standard reference test

n = 208

**Negative** to the standard reference test

n = 133

**Final diagnosis**

- Infected with *T. cruzi* (n = 0)
- Not infected with *T. cruzi*

(n = 133)

- Indeterminate (n = 0)

**Final diagnosis**

- Infected with *T. cruzi*

(n = 0)

- Not infected with *T. cruzi*

(n = 0)

- Indeterminate (n = 0 )

**Final diagnosis**

- Infected with *T. cruzi*

(n = 208)

- Not infected with *T. cruzi*

(n = 1)

- Indeterminate (n = 0)
